# Population PK Modeling of Denosumab Biosimilar MB09 and Reference Denosumab to Establish PK Similarity

**DOI:** 10.3390/pharmaceutics17091146

**Published:** 2025-09-01

**Authors:** Sara Sánchez-Vidaurre, Alexandra Paravisini, Javier Queiruga-Parada

**Affiliations:** Medical Department, mAbxience Research S.L., 28050 Madrid, Spain

**Keywords:** biosimilar, denosumab, MB09, meta-analysis, pharmacokinetics, population modeling

## Abstract

**Background/Objectives**: MB09 is a denosumab biosimilar to the reference products (RPs) Xgeva and Prolia. A population pharmacokinetic (popPK) meta-analysis was conducted to characterize the denosumab PK profile and to support MB09 biosimilarity. **Methods**: Pooled denosumab PK data from one phase I study [255 healthy adult men receiving a single 35 mg subcutaneous (SC) dose] and one phase III study (555 postmenopausal women with osteoporosis receiving two 60 mg SC doses, one every six months) were used. A one-compartment model with first-order absorption and elimination and parallel non-linear saturable clearance was used. Body weight was included on clearance as a structural covariate and treatment was tested as a covariate on all PK parameters. PK biosimilarity was assessed at 35 mg dose. **Results**: For a 70 kg subject, the apparent clearance and central volume of distribution for denosumab were 0.123 L/day [95% confidence interval (CI): 0.114, 0.132] and 9.33 L (95% CI: 9.11, 9.55), respectively. The Michaelis constant was 0.124 ng/mL and the maximum rate for the non-linear clearance was 0.139 ng/day. Model-based bioequivalence criteria were met for RP Xgeva, European and US-sourced, versus MB09 for a dose of 60 mg SC. The mean area under the plasma concentration curve (AUC) resultant from the simulation of MB09 120 mg SC was similar to the published mean AUC observed for Xgeva 120 mg SC every four weeks. **Conclusions**: This analysis provides a valuable assessment of denosumab PK characteristics and elucidates in more detail how the MB09 PK profile compares to the denosumab RPs, supporting the totality of evidence on MB09 biosimilarity.

## 1. Introduction

Denosumab is a human monoclonal immunoglobulin G2 antibody that was first approved in 2010 for the treatment and prevention of bone loss indications and bone complications in advanced cancers under the brand name of Prolia^®^ and Xgeva^®^ (Amgen Europe B.V., Breda, The Netherlands) [1,2]. Denosumab binds with high affinity and specificity to the receptor activator of nuclear factor kappa-B (RANK) ligand (RANKL) and prevents activation of RANK on the surface of osteoclast precursors and osteoclasts. This inhibition prevents the development of osteoclasts, the cells responsible for bone resorption which play a critical role in bone modeling and remodeling [3]. The two denosumab reference products (RPs) have different indications and different dosage strengths. Prolia^®^ is formulated as a 60 mg/mL solution to be administered subcutaneously (SC) by means of a single-dose prefilled syringe every six months, while Xgeva^®^ is formulated as a 70 mg/mL solution in a single-dose vial to be administered SC mainly in a 120 mg dose every four weeks

MB09 is a biosimilar to both denosumab RPs developed by mAbxience Research S.L. The MB09 clinical development program included two studies: a phase I study for pharmacokinetic (PK) similarity evaluation and a phase III confirmatory study for therapeutic equivalence assessment. The phase I study (MB09-A-01-19) aimed to compare the PK/pharmacodynamic (PD), safety and immunogenicity profile of MB09 to those of European Union (EU)- and United States of America (US)-sourced Xgeva^®^ (Amgen Inc., Thousand Oaks, CA, USA) (NCT05299073) [4]. This study, conducted in healthy volunteers, demonstrated similar PK, PD, safety and immunogenicity characteristics between MB09 and Xgeva^®^. The phase III study (MB09-C-01-19) was conducted in postmenopausal women with osteoporosis and had the primary objective of comparing the efficacy of MB09 and Prolia^®^ sourced from the EU (EU-Prolia), and the secondary objectives included PK, PD, safety and immunogenicity assessments between both products (NCT05338086). Study results demonstrated equivalent clinical performance of MB09 and Prolia^®^.

Denosumab PK profile is non-linear below doses of 60 mg, where elimination is primarily specific target-mediated. At doses of 60 mg and above, the PK profile becomes approximately linear, the target-mediated clearance (CL) is negligible and exposure increases are dose-proportional. After SC administration, bioavailability is ~61%, with maximum observed concentration (C_max_) reached in 10 days and a terminal half-life of 25–32 days, supporting dosing every six months (60 mg dose) or every four weeks (120 mg dose). Denosumab is cleared via reticuloendothelial catabolism, not renal or hepatic pathways, and shows no significant accumulation or PK alterations due to age, gender, race or mild-to-moderate organ impairment [1,5]. CL and central volume of distribution (V) appear proportional to body weight [1]. Although a higher body weight is associated with lower exposure, this is not considered clinically important as PD effects based on bone turnover markers and bone mineral density (BMD) increases are consistent across a wide range of body weights [5].

Population PK (popPK) modeling quantitatively characterizes drug concentration-time profiles and variability within a population using clinical data and has become an essential tool in biosimilar development for demonstrating PK equivalence between biosimilar candidates and RPs. As a new approach methodology (NAM), popPK modeling leverages existing data to provide robust comparative evidence, reducing the need for additional animal or healthy volunteer studies and minimizing unnecessary drug exposure in the efficacy/safety assessment of new medicines [6]. In this context, the present popPK meta-analysis was conducted to characterize the PK profile of MB09 and RP Xgeva^®^ in healthy subjects and individuals with osteoporosis to assess the influence of demographic covariates, and to investigate potential differences between approved denosumab dosing regimens. This approach not only supports regulatory decision-making for biosimilar development but also aligns with current ethical and scientific standards in clinical pharmacology.

## 2. Materials and Methods

### 2.1. Study Design and Subjects

PK data from the phase I study in healthy male subjects and the phase III study in women with postmenopausal osteoporosis were pooled and used to build a non-linear mixed effect modeling (NONMEM) input dataset which contained serum concentration-time data for building the popPK model.

A total of 810 (255 healthy subjects and 555 postmenopausal women with osteoporosis) subjects who received denosumab by SC administration were used for this popPK analysis (Table 1).

The phase I study was a randomized, double-blind, three-arm, single-dose, parallel study conducted in healthy men volunteers who were randomly assigned in a 1:1:1 ratio to receive either 35 mg SC dose of MB09, EU-Xgeva or US-Xgeva. The phase III study was a randomized, double-blind, parallel, multicenter, multinational study where postmenopausal women were randomly assigned in a 2:1:1 ratio to one of the three study arms: arm 1 MB09-MB09, to receive one SC injection (60 mg/mL) of MB09 on Day 1, Month 6, and Month 12; arm 2 Prolia-MB09, to receive one SC injection of EU-Prolia on Day 1 and at Month 6 and one SC injection of MB09 at Month 12; arm 3 Prolia-Prolia, to receive one SC injection of EU-Prolia on Day 1, Month 6, and Month 12.

Study protocols were approved by the responsible local Independent Ethics Committee or Institutional Review Board. These studies were conducted in accordance with the principles of the International Council for Harmonisation of Technical Requirements for Pharmaceuticals for Human Use harmonised tripartite guideline E6(R2): Good Clinical Practices, the ethical principles of the Declaration of Helsinki and all applicable regulations. A written informed consent form was signed by each subject before the study enrolment.

Serum samples for the PK assessment were collected at prespecified time points in each of the studies (Table 1). In both studies, denosumab serum concentrations were quantitatively determined using a validated Meso Scale Discovery^®^ (MSD, Frederick, MD, USA) electrochemiluminescence assay. Values below the limit of quantification (LLOQ) at the beginning of a given profile were imputed with zero. Values at the end of a profile (i.e., after the last incidence of a measurable concentration) and individual samples below LLOQ which fell between two measurable concentrations were omitted from the analysis.

### 2.2. Data Analysis

PopPK analyses were carried out using NONMEM version 7.4.3 with PREDPP, PDx-Pop version 5.2.2 (Icon Development Solutions, Ellicott City, MD, USA) and Compaq Visual Fortran Optimizing Compiler version 12 on Microsoft Windows 10 platform (Microsoft, Redmond, WA, USA).

The model was fit using the first order conditional estimation method available in NONMEM and THETAs representing typical PK parameters on the natural log scale.

Output tables were post-processed, and diagnostic plots and covariate investigation were conducted using R version 3.6.2 (R Foundation for Statistical Computing, Vienna, Austria). Final models were evaluated by visual predictive check (VPC).

Successful runs were based on the methodology used for fitting the models, but the following were also key considerations: successful model convergence, non-singular covariance matrix and completion of the covariance step without warnings.

For optimal model selection, the bespoke diagnostic plots during the preliminary runs were evaluated at each stage of model development. The standard criteria of change in the minimum objective function value (OFV) and review of the following diagnostics were also carried out when appropriate for the analyses: plots of DV (observed concentration value) versus PRED (population predicted concentration value); plots of DV versus IPRE (individual predicted concentration values); plots of CWRES (conditional weighted residuals) versus PRED; plots of CWRES versus IPRE; plots of CWRES versus time; population parameter estimates that were reasonable and supported in the context of the structural model, percent relative standard error (%RSE), and 95% confidence interval (CI) for the parameter estimates; value of the objective function (smaller is better).

### 2.3. PK Modeling Development

Initially, the PK study rich PK data were modeled. Treatment was included as a covariate on all PK parameters sequentially in pairwise comparisons. Subsequently, the sparse PK data from the efficacy study were added, and the model was re-evaluated.

#### 2.3.1. Base PK Model

The denosumab PK profile has previously been described with popPK models using quasi steady-state approximation of the target-mediated drug disposition where RANKL levels were also available [7,8]. With no RANKL levels available, the non-linear saturable process was described with a Michaelis–Menten term [9].

A one-compartment popPK model with first-order absorption and elimination parameterized in terms of CL, V and absorption rate constant (KA) was fitted to denosumab serum concentration data. CL was described by a parallel process with one linear component (non-specific elimination) and a non-linear, saturable component (target-mediated elimination) described by a Michaelis–Menten term.

The selection of the one-compartment model was based on both empirical and physiological considerations. Model selection was guided by comparison of objective function values and information criteria (AIC, BIC) across alternative structural models, including two-compartment models. The one-compartment model provided an adequate fit to the observed data, as confirmed by goodness of fit diagnostics and VPCs. More complex models did not yield significant improvements in fit or predictive performance. The one-compartment model choice was further supported by the known PK properties of denosumab, which, as a monoclonal antibody, exhibits limited extravascular distribution and an V approximating plasma volume [1,10]. Thus, the one-compartment model with parallel linear and non-linear elimination was considered both scientifically and physiologically appropriate for describing denosumab PK profile in the present popPK meta-analysis.

#### 2.3.2. Variability Models

##### Model for Inter-Individual Variability

The MU modeling technique was utilized in NONMEM [11]. Following this approach, MU was set equal to THETA. A particular model parameter (P_i_) was set equal to EXP(MU + ETA(i)).

P_i_ is the estimated parameter for the ith individual and in the case of CL:

MU_1 = THETA(1)(1)

CL/F = EXP(MU_1 + ETA(1)).(2)

THETA (and MU) is the population value for the parameter on the natural log scale. ETA(i) is inter-individual random effect for the ith subject and parameter P, and is distributed as follows: ETA(i)*~N*(0, ω^2^) (i.e., a normal distribution with mean of zero and variance of ω^2^).

##### Model for Residual Variability

A proportional error model was selected for most terms to characterize residual variability in the final popPK model. Residual variability (ε), which is the difference between the observed and the individual predicted observations, was described as the following in the final popPK model:

*C*_ij_ = ^*C*_ij_ + ε_pij_ (3)

where *C*_ij_ is the jth observation in the ith individual.; ^*C*_ij_ is the jth predicted value in the ith individual; and ε_pij_ is residual random error for the jth observation in the ith individual and is distributed as follows: ε~*N*(0, σ^2^).

#### 2.3.3. Covariate Analysis

PK model development included the investigation of demographic covariates known to affect serum denosumab PK. The list of covariates tested included body weight, which was evaluated for the apparent clearance (CL/F), and treatment, which was evaluated for CL/F, apparent volume of distribution in the central compartment (V/F) and KA.

For the covariate modeling, investigation of covariate–parameter relationships was based on the range of covariate values in the dataset, scientific interest, mechanistic plausibility and exploratory graphics. Prior to including covariates in the population model, visual inspection of the relationship between each ETA (random effect term for a parameter) and the covariates was performed using scatterplots. Covariates that appeared to be correlated with an ETA through the visual inspection were formally evaluated. Centering of covariates was considered, as appropriate, for continuous covariates. In the current analysis, the continuous covariates were centered to 70 kg.

### 2.4. Model Evaluation and Model-Based Bioequivalence

A VPC was performed using the parameter estimates obtained in the final modeling step including the random effects. The predicted values were estimated using the complete NONMEM dataset. Five hundred simulations from the popPK model were used for creating the VPC figures. A sensitivity analysis comparing one- and two-compartment models was performed. Due to convergence issues with the two-compartment model, the one-compartment model, which adequately fit the majority of subjects, was selected.

Derived PK parameters C_max_, AUC_0-τ_ and AUC_∞_ based on the individually predicted denosumab concentrations from the final model were calculated with Phoenix WinNonlin^TM^ version 8.3 (Certara Inc., Princeton, NJ, USA).

### 2.5. Simulations

Simulations were performed to support clinical extrapolation to doses of 120 mg, the therapeutic dose for cancer indications not covered by the doses employed in the two clinical studies, and were compared to results available in the public domain.

The final models were used to simulate PK profiles from a multiple dosing of MB09 versus the RP to evaluate impact on the PK in different patient populations.

Individual-level AUC and C_max_ values from the simulations were not retained in a format suitable for distributional analysis; therefore, only summary statistics [mean and standard deviation (SD)] were calculated.

## 3. Results

### 3.1. Subjects Characteristics

The PK analysis included 255 healthy men (85 subjects per treatment arm) and 555 postmenopausal women with osteoporosis (278 subjects received MB09 and 277 subjects received Prolia), contributing with 4256 (phase I study) and 3026 (phase III study) PK samples, respectively. Summary of demographics and baseline characteristics for subjects in the popPK analysis is provided for each study in Table 2.

### 3.2. Modeling Results

#### 3.2.1. PK Model Phase I Study

**Figure 1:**
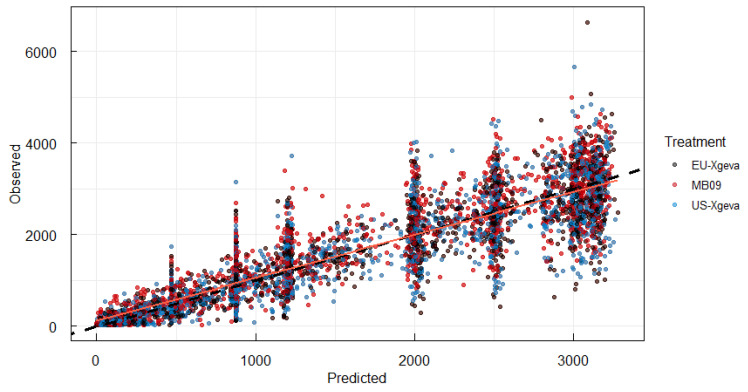
Predicted versus observed denosumab concentration. Y-axis and X-axis represent denosumab serum concentration (unit = ng/mL). Solid line is a fitted line through data; dashed line is the line of unity.

To start, a popPK model was built using only data from the phase I study. A one-compartment model with first-order absorption and elimination and parallel non-linear saturable CL, parameterized in terms of CLs and volumes, was initially used to describe denosumab serum PK characteristics. The model was fit to non-transformed PK data with the ADVAN13 NONMEM subroutine and FOCE method with interaction using NONMEM version 7.4.3. All parameters were log-transformed using MU referencing technique before estimation. Between-subject variability was included for PK parameters CL/F, V/F and KA as exponential random effects. A proportional residual error model was used to describe intra-individual variability. Model goodness of fit, demonstrated by conventional diagnostic plots, was adequate (Figure 1).

Investigation into the use of additive, proportional, and additive/proportional error was tested to determine the impact on serum concentration estimation. The proportional error term was chosen. To further improve prediction of disposition, a non-linear CL term was added and described as a Michaelis–Menten process, which considerably improved the fit. Gender and age were not tested formally as covariates because both could be confounded with the study. Body weight was found significant and included as a structural covariate. Regarding anti-drug antibodies, only 10 subjects (4 in the phase I study and 6 in the phase III study) out of 810 presented positive samples and, due to this low incidence, the impact of anti-denosumab antibodies on the model was dismissed. Results of the popPK model with phase I study are presented in Table 3.

#### 3.2.2. Comparison of Treatments

Subsequently, treatment was tested as a covariate on all PK parameters. All PK parameters with inter-individual variability had shrinkages <10% [12].

Pairwise comparisons were made to include data from MB09, EU-Xgeva and US-Xgeva from the phase I study. There was no significant improvement in the fit when treatment was tested as a covariate on the PK parameters in the three pairwise comparisons. The boxplots of PK parameters by treatment showed no difference between MB09, EU-Xgeva and US-Xgeva (Supplementary Figure S1).

#### 3.2.3. PK Model Both Studies

A one-compartment model with the same characteristics as the PK Model phase I study was used to describe denosumab serum PK. Body weight was included on CL as a structural covariate as previously. Possible differences between the PK parameters (CL/F, V/F) were investigated by testing the study as a covariate. The study on V/F improved the fit and was included in the final model. The parameter estimates for the final popPK model are presented in Table 4.

### 3.3. Model Evaluation Results

#### 3.3.1. Visual Predictive Check

**Figure 2:**
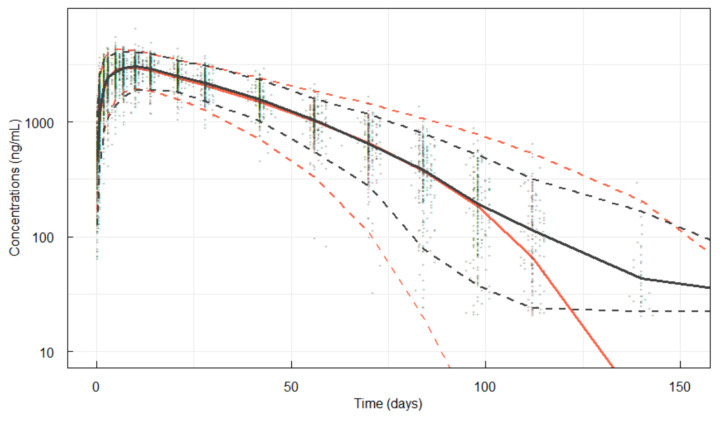
VPC for serum denosumab versus time following a single 35 mg dose in healthy subjects (Run808). Red lines are 5, 50 and 95th percentiles of the simulations from the model. Black lines are 5, 50 and 95th percentiles of the observations; black markers are the observations.

**Figure 3:**
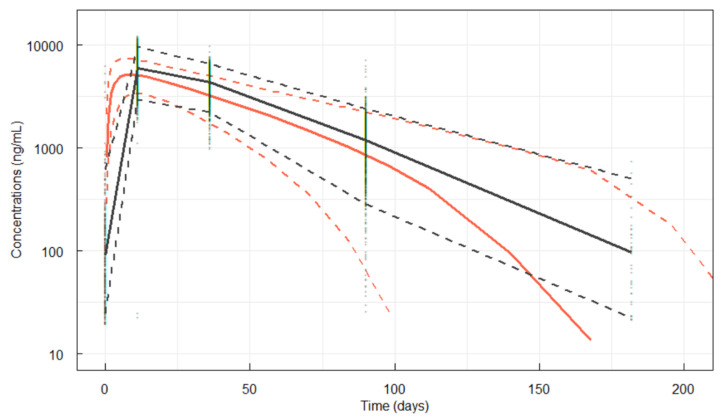
Simulations for denosumab PK following a single 60 mg dose. Red lines are 5, 50 and 95th percentiles of the simulations from the model. Black lines are 5, 50 and 95th percentiles of the observations; black markers are the observations.

As a VPC, serum denosumab concentrations were simulated (the complete study dataset was simulated 500 times). These data were overlaid with the observed PK concentration data. The regimen evaluated was a single 35 mg dose of denosumab (Figure 2) and a single 60 mg dose of denosumab (Figure 3). The VPC plots showed good overall fit of the simulation to the observed data.

#### 3.3.2. Sensitivity Analysis

Five subjects (two subjects receiving MB09, two subjects receiving EU-Xgeva and one subject receiving US-Xgeva) in the phase I study had measurable denosumab samples at late time points (time > 140 days). For those subjects, a two-compartment model would describe better their concentration-time profiles. A sensitivity analysis was performed by excluding those subjects to inspect the impact of the misspecification from them on the overall model. A side-by-side comparison of the PK parameter estimates demonstrated that the impact was marginal (Supplementary Table S1).

### 3.4. Model-Based Bioequivalence

The individual predictions of denosumab concentrations from the final model were used to calculate the derived PK parameters C_max_, AUC_0-τ_ and AUC_∞_ (Supplementary Table S2); afterwards, the ratios of both RPs versus MB09 were calculated. In all instances, the upper and lower 90% CIs for the three derived PK parameters fell well within the pre-defined margin (80–125%) for biosimilarity when comparing MB09 to both Eu-Xgeva and US-Xgeva (Supplementary Table S3).

### 3.5. Simulations

Simulations of the final model after a single 60 mg denosumab dose and observations from the phase III study are presented in Figure 3.

**Figure 4:**
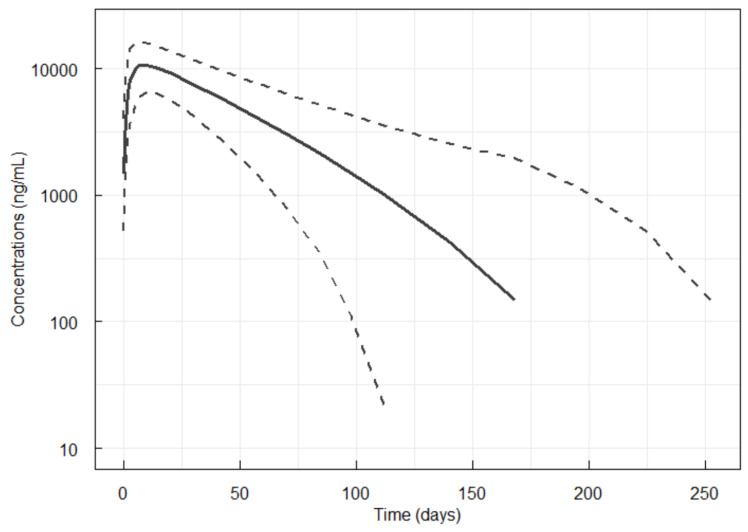
Simulations for denosumab PK following a single 120 mg dose. Black lines are 5, 50 and 95th percentiles of the observations.

Simulations with a single SC dose of 120 mg denosumab were performed using all data and the PK model of both studies to ensure coverage of a wide range of demographics. These simulations are presented in Figure 4.

Finally, the mean AUC_0-∞_ was calculated for the simulations following a single 120 mg SC dose of denosumab and resulted in 753,000 (SD: 285,200).

## 4. Discussion

This study presents a popPK meta-analysis of denosumab PK data across two clinical studies involving over 800 subjects. PK data were well-described by a one-compartment model with first-order absorption, parallel first-order, saturable elimination and body weight on CL. No significant differences were observed in denosumab PK concentration-time profiles after treatments with a single dose of 35 mg for the three pairwise comparisons. Model-based bioequivalence criteria were met for MB09 versus both RPs.

When investigating the PK characteristics of monoclonal antibodies, V and/or elimination are often reported to be influenced by body weight, although it is not always clinically significant [13,14]. For denosumab, in a previous popPK meta-analysis including a similar study population to the one used in the present popPK meta-analysis, both CL and Vwere proportional to body weight [7]. This relationship is consistent with the principles of allometry, which describe how biological variables scale with body size, and according to these principles, the effect of body weight should preferably be added to CL as it accounts for both body size and differences in metabolic rates across size [15]. This approach was used in the present study and ensures that the PK model accurately reflects the physiological relationship between body weight and drug disposition, leading to a more robust prediction of drug exposure across different body sizes.

The present study did not formally test the effect of age or gender due to potential confounding with the effects of study. According to the popPK meta-analysis by Sutjandra et al., the effects of age on the AUC of denosumab were less than 15% over the range of covariate values that were evaluated, which included age [7]. This suggests that the denosumab PK profile is relatively consistent across different age groups, including subjects younger than 60 years and those aged 60–80 years.

Denosumab immunogenicity was observed to be very low in this study as ADA positive samples were only detected in 10 out of 810 subjects, which is in line with what has been reported for both Prolia^®^ and Xgeva^®^ [1,5,16] and for others denosumab biosimilars [17,18]. The low incidence of ADA positivity observed in this study supports the favorable safety profile of denosumab and its very low risk of immunogenicity. Nevertheless, a recent study of a denosumab biosimilar found higher rates of ADAs as a result of the high sensitivity of the ADA assay used, but most observed ADAs were transient in nature [19].

PK similarity was assessed in this study at the most sensitive dose, 35 mg, where the non-linear target mediated process is most discernible. Subsequently, addition of sparse PK data after 60 mg of denosumab enabled building a popPK model that could be confidently used to extrapolate and simulate the MB09 PK after a 120 mg dose. This extrapolation is considered adequate due to the nearly dose-linear behavior observed at doses above 60 mg, where the denosumab PK parameters, including AUC and C_max_, increase in a dose-proportional manner [1,20]. This fact is because the RANKL mediated pathway is almost fully saturated and denosumab disposition is mainly driven by the linear elimination (non-saturable) pathway [7]. The results obtained in the present study on the simulations with the 120 mg SC dose showed very good correspondence, by visual inspection, with previously published data by Sutjandra et al. where the authors characterized the non-linear PK profile of denosumab through model-based simulations [7]. From the results obtained in the present study where MB09 demonstrated to be bioequivalent to Xgeva at the most sensitive dose, it can be assumed that the bioequivalence also holds at 120 mg where the denosumab PK is dose-linear.

Additionally, the mean AUC obtained with the 120 mg simulations can be compared to the published observed AUC in cancer patients (AUC_0-τ_ at steady-state reported to be 723,000 (SD: 684,000) ng·h/mL) [21]. AUC values are similar and the observed 2-fold variability in AUC in oncology patients compared to the simulations can be attributed to differences in health status of the subjects included in the two studies, where the systematic review by Sohn et al. included data from 116 healthy subjects and 592 patients with advanced cancer and bone metastases. Factors such as disease state, concomitant medications and overall health condition can influence drug metabolism and distribution, leading to a greater inter-subject variability in the PK parameters. However, data from cancer patients indicate that the denosumab PK levels are consistent with data from healthy adults.

A limitation of the present analysis is that individual-level AUC and C_max_ values from the simulations were not retained in a format suitable for distributional analysis, precluding the generation of box plots or other visualizations of their distributions. Nevertheless, the use of summary statistics and comparative analyses provides a robust assessment of clinical extrapolation to higher doses not tested in the clinical studies.

One strength of this popPK meta-analysis is the large sample size of subjects, including healthy subjects and women with postmenopausal osteoporosis. In addition, the inclusion of healthy subjects allows a better comprehensiveness of the model and results in a robust description of the denosumab PK profile.

In conclusion, this popPK meta-analysis provides a valuable assessment of denosumab PK characteristics and elucidates in more detail how the PK compares between the denosumab biosimilar and the RP. MB09 PK bioequivalence to reference denosumab is demonstrated in a pooled population of healthy subjects and postmenopausal women with osteoporosis, supporting the totality of evidence on MB09 biosimilarity.

## Figures and Tables

**Table 1:** Overview of clinical studies included in the popPK meta-analysis.

Study	Study Description/Phase	Dose(s)/Frequency/Treatment	Scheduled Sampling Timepoints
MB09-A-01-19 PK study	A randomized, double-blind, three-arm, single-dose, parallel study to compare the pharmacokinetics, pharmacodynamics, safety and immunogenicity profile of MB09 (denosumab biosimilar) and EU/US-sourced Xgeva^®^ in healthy male volunteers	35 mg (n = 255) Single SC dose MB09 (TRT = 1) EU-sourced Xgeva (TRT = 2) US-sourced Xgeva (TRT = 3)	Serum: Rich PK Pre-dose, 8 and 16 h (±2 h), 24, 48, and 72 h (±4 h), Days 6, 8, and 11 (±1 day), Days 15, 22, and 29 (±2 days), Days 43, 57, 71, 85, 99, 113, 141, 169, 197, 225, and 253 (±3 days)
MB09-C-01-19 Efficacy study	A randomized, double-blind, parallel, multicenter, multinational study to compare the efficacy, pharmacokinetics, pharmacodynamics, safety and immunogenicity of MB09 (denosumab biosimilar) versus Prolia^®^ (EU-sourced) in postmenopausal women with osteoporosis (SIMBA Study)	60 mg (n = 555) 3 SC doses, every 6 months MB09 (TRT = 1) EU-sourced Prolia (TRT = 2)	Serum: Sparse PK Day 1 (0 predose), Day 11 and at Month 1, Month 3, Month 6 (predose) and Month 12 (predose) in the main period

Abbreviations: EU, European Union; PK, pharmacokinetics; SC, subcutaneous; TRT, treatment; US, United States.

**Table 2:** Summary of subject characteristics at baseline.

	MB09-A-01-19 Study (*N* = 255)	MB09-C-01-19 Study (*N* = 555)
Age (years)		
Mean (SD)	39.5 (6.90)	65.8 (5.94)
Age group, *n* (%)		
≥28 to <55 years	254 (99.608)	0 (0.0)
≥55 to <68 years	1 (0.392)	342 (61.6)
≥68 to ≤80 years	0 (0.0)	213 (38.4)
Smoking status, *n* (%)		
Current Smoker	0 (0.0)	132 (23.8)
Former Smoker	0 (0.0)	74 (13.3)
Never Smoked	0 (0.0) *	349 (62.9)
Race, *n* (%)		
White	255 (100.0)	551 (99.3)
American Indian or Alaska Native	0 (0.0)	4 (0.7)
Ethnicity, *n* (%)		
Hispanic or Latino	0 (0.0)	23 (4.1)
Not Hispanic or Latino	255 (100.0)	532 (95.9)
Height (cm)		
Mean (SD)	178.66 (6.227)	159.98 (6.186)
Weight (kg)		
Mean (SD)	82.97 (8.492)	63.196 (8.7870)
Body mass index (kg/m^2^)		
Mean (SD)	25.98 (2.396)	24.683 (3.0401)

* Exclusion criterium: no tobacco within one year previous to the study, but no other smoking status registered. Abbreviations: SD, standard deviation.

**Table 3:** PopPK parameters of PK model phase I study.

Parameters (Units)	Final Estimate	% RSE	95% CI		Inter-Individual Variability
			Lower	Upper	
KA (1/day)	0.406	3.92%	0.375	0.437	61%
V/F (L)	9.33	1.21%	9.11	9.55	13%
CL/F (L/day)	0.123	3.55%	0.114	0.132	37%
BW~CL	1.32	16.2%	0.901	1.74	
Km (ng/mL)	0.124	9.27%	0.101	0.147	
Vm (ng/day)	0.139	5.28%	0.125	0.153	
Residual variability					17%

Abbreviations: BW, body weight; CI, confidence interval; CL, clearance; CL/F, apparent systemic clearance; KA, absorption rate constant; Km, Michaelis–Menten constant; RSE, relative standard error; V/F, apparent volume of distribution; Vm, maximum rate of metabolism.

**Table 4:** PopPK parameters of final PK model, both studies.

Parameters (Units)	Final Estimate	%RSE	95% CI		Inter-Individual Variability
			Lower	Upper	
KA (1/day)	0.349	2.7%	0.330	0.368	60%
V/F (L)	9.00	0.8%	8.86	9.14	15.7%
CL/F (L/day)	0.143	2.6%	0.136	0.150	34.6%
BW~CL	1.07	9.8%	0.864	1.28	
Km (ng/mL)	0.162	18.8%	0.102	0.222	
Vm (ng/day)	0.128	7.6%	0.109	0.147	
Study~V	−0.534	33.7%	−0.887	−0.181	
Residual variability					19.5%

Abbreviations: BW, body weight; CI, confidence interval; CL, clearance; CL/F, apparent systemic clearance; KA, absorption rate; Km, Michaelis–Menten constant; RSE, relative standard error; V, volume; V/F, apparent volume of distribution; Vm, maximum rate of metabolism.

## Data Availability

The datasets generated and/or analyzed during the current study are available from the corresponding author upon reasonable request.

## Supplementary Materials

The following supporting information can be downloaded at: https://www.mdpi.com/article/10.3390/pharmaceutics17091146/s1, Figure S1: Covariate plots for PK model; Table S1: Sensitivity analysis—PopPK parameters comparison; Table S2: Summary statistics of the model-based derived PK parameters; Table S3: Statistical comparison of the model-based derived PK parameters.
