# Supplementary material for: Population PK Modeling of Denosumab Biosimilar MB09 and Reference Denosumab to Establish PK Similarity
Source: Pharmaceutics. 2025 Sep 1;17(9):1146. doi: 10.3390/pharmaceutics17091146 (PMC12473923; doi:10.3390/pharmaceutics17091146)
Supplement: Supplementary file 1 [file pharmaceutics-17-01146-s001.zip › pharmaceutics-3794947-supplementary.pdf]

Article

Population PK Modeling of Denosumab Biosimilar MB09 and Reference Denosumab to Establish PK Similarity

Sara Sánchez-Vidaurre[1], Alexandra Paravisini[1] and Javier Queiruga-Parada[1,*]

[1] Medical Department, mAbxience Research S.L., Madrid 28050, Spain; sara.sanchezv@mabxience.com (S.S.-V.); alexandra.paravisini@mabxience.com (A.P.)
* Correspondence: javier.queiruga@mabxience.com (J.Q.-P.)

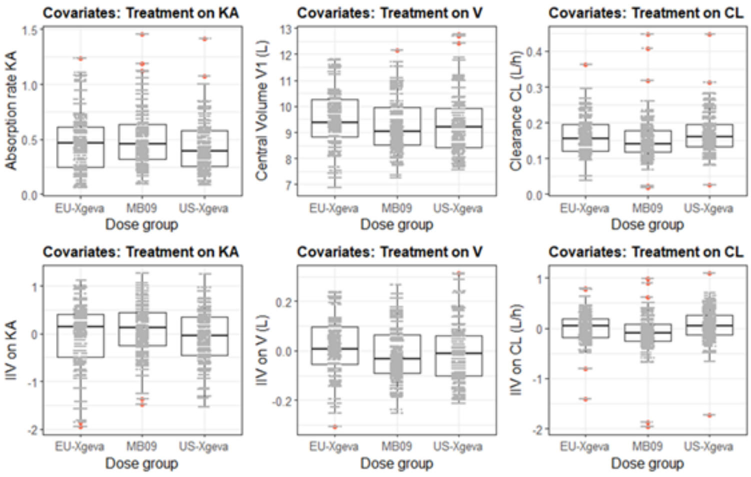

Abbreviations: CL, clearance; KA, absorption rate constant; pK, pharmacokinetics; V, central volume of distribution.

Supplemental Figure 1. Covariate plots for PK model.

Supplemental Table 1. Sensitivity analysis – PopPK parameters comparison.

PK Parameter	All Subjects. Estimate (RSE%)	5 Subjects Excluded. Estimate (RSE%)
KA (1/day)	0.406 (3.92%)	0.420 (3.86%)
V (L)	9.33 (1.21%)	9.53 (1.43%)
CL (L/day)	0.123 (3.55%)	0.110 (6.10%)
Km (ng/mL)	0.124 (9.27%)	0.164 (10.7%)
Vm (ng/day)	0.139 (5.28%)	0.178 (7.25%)

Abbreviations: CL, clearance; KA, absorption rate constant; Km, Michaelis-Menten constant; popPK, population pharmacokinetic; RSE%, relative standard error percentage; V, central volume of distribution; Vm, maximum rate of metabolism.

Supplemental Table 2. Summary statistics of the model-based derived PK parameters

Treatment	Parameter	Units	N	Geometric Mean	Geometric SD	CV %	Min	Median	Max	SD
EU-Xgeva	$AUC_{0\text{-}6Months}$	day*ng/mL	85	139477	1.26	22.94	69192	139355	255897	32833
	AUC_{∞}	day*ng/mL	85	139635	1.26	23.34	69185	139353	266451	33458

Treatment	Parameter	Units	N	Geometric Mean	Geometric SD	CV %	Min	Median	Max	SD
	C_{max}	ng/mL	85	3020	1.21	17.20	1478	3095	4307	528
MB09	$AUC_{0-6Months}$	day*ng/mL	85	148086	1.30	25.88	63699	150866	310099	39600
	AUC_{∞}	day*ng/mL	85	148433	1.30	27.29	63681	150861	336577	41928
	C_{max}	ng/mL	85	3188	1.16	14.52	1869	3243	4124	468
US-Xgeva	$AUC_{0-6Months}$	day*ng/mL	85	135946	1.28	24.38	62434	139253	257949	34137
	AUC_{∞}	day*ng/mL	85	136115	1.28	24.98	62424	139245	276867	35044
	C_{max}	ng/mL	85	3069	1.20	17.43	1874	3156	4280	544

Abbreviations: $AUC_{0-6Months}$, area under the concentration-time curve from time 0 to 6 months; AUC_{∞}, area under the concentration-time curve extrapolated to infinity; C_{max}, maximum observed concentration; CV%, coefficient of variation percentage; EU, European Union; Max, maximum value observed; Min, minimum value observed; N, number of subjects; PK, pharmacokinetic; SD, standard deviation; US, United States.

Supplemental Table 3. Statistical comparison of the model-based derived PK parameters

		Ln($AUC_{0-\tau}$)		Ln(AUC_{∞})		Ln(C_{max})	
**Reference**	**Test**	**CI90_Lower**	**CI90_Upper**	**CI90_Lower**	**CI90_Upper**	**CI90_Lower**	**CI90_Upper**
EU-Xgeva	MB09	99.75	113.0	99.77	113.25	101.07	110.29
US-Xgeva	MB09	102.14	116.17	102.15	116.42	99.55	108.45

Abbreviations: CI90, 90% confidence interval; EU, European Union; Ln($AUC_{0-\tau}$), natural logarithm of the area under the concentration-time curve over the dosing interval; Ln(AUC_{∞}), natural logarithm of the area under the concentration-time curve extrapolated to infinity; Ln(C_{max}), natural logarithm of the maximum observed concentration; PK, pharmacokinetic; US, United States.
